# Factors associated with electrocardiographic left ventricular hypertrophy among patients with hypertension in Thailand

**DOI:** 10.1186/s40885-024-00267-8

**Published:** 2024-04-01

**Authors:** Boonsub Sakboonyarat, Jaturon Poovieng, Ram Rangsin

**Affiliations:** 1 Department of Military and Community Medicine, Phramongkutklao College of Medicine, Bangkok, Thailand; 2 Pulmonary and Critical Care Division, Department of Medicine, Phramongkutklao College of Medicine, Bangkok, Thailand

**Keywords:** Left ventricular hypertrophy, Hypertension, Tobacco use, Thailand

## Abstract

**Background:**

Left ventricular hypertrophy (LVH) strongly predicts cardiovascular diseases (CVD) and death. One-fourth of Thai adults suffer from hypertension. Nevertheless, the information on LVH among Thai patients with hypertension is not well characterized. We aimed to identify the prevalence and factors associated with electrocardiographic LVH (ECG-LVH) among patients with hypertension in Thailand.

**Methods:**

The present study obtained the dataset from the Thailand Diabetes Mellitus/Hypertension study, which included hypertension patients aged 20 years and older receiving continuous care at outpatient clinics in hospitals nationwide in 2011–2015 and 2018. Meanwhile, those without a record of 12-lead electrocardiography (ECG) were excluded from the analysis. ECG-LVH was defined as the LVH noted regarding ECG interpretation in the medical records. Multivariable logistic regression analysis was utilized for determining factors associated with ECG-LVH and presented as the adjusted odds ratio (AOR) and 95% confidence interval (CI).

**Results:**

From 226,420 hypertensive patients in the Thailand Diabetes Mellitus/Hypertension study, 38,807 individuals (17.1%) with ECG data recorded were included in the analysis. The mean age was 64.8 ± 11.5 years, and 62.2% were women. Overall, 1,557 study participants had ECG-LVH, with an estimated prevalence of 4.0% (95% CI, 3.8–4.2%). Age-adjusted ECG-LVH prevalence among women and men was 3.4 and 5.1%, respectively (*P* < 0.001). Multivariable analysis determined factors associated with ECG-LVH, including being men (AOR, 1.49; 95% CI, 1.31–1.69), individuals aged 70 to 79 years (AOR, 1.56; 95% CI, 1.20–2.02) and ≥ 80 years (AOR, 2.10; 95% CI, 1.58–2.78) compared to individuals aged less than 50 years, current smokers (AOR, 1.26; 95% CI, 1.09–1.46) compared to those who never smoked, systolic blood pressure ≥ 140 mmHg and diastolic blood pressure ≥ 90 mmHg (AOR, 1.58; 95% CI, 1.30–1.92) compared to systolic blood pressure < 140 mmHg and diastolic blood pressure < 90 mmHg.

**Conclusions:**

The current study illustrated the prevalence of ECG-LVH among Thai patients with hypertension who had ECG recorded and identified high-risk groups who tended to have ECG-LVH. The findings underscore the need for targeted interventions, particularly among high-risk groups such as older individuals, men, and current smokers, to address modifiable factors associated with ECG-LVH.

**Supplementary Information:**

The online version contains supplementary material available at 10.1186/s40885-024-00267-8.

## Background

Left ventricular hypertrophy (LVH) is a medical condition that involves an increase in the mass of the myocardium of the left ventricle of the heart. This thickening of the heart muscle occurs as a response to prolonged elevated workload on the heart, but it is considered abnormal and pathological in the long run [1]. LVH is a manifestation of hypertensive target organ damage and significantly predicts the occurrence of cardiovascular diseases (CVD), including ischemic heart disease (IHD), stroke, heart failure, and sudden cardiac death [2–5]. A large cohort study in the United States with an average follow-up period of 7 years has revealed that among 140,387 individuals diagnosed with hypertension, the incidence of new-onset electrocardiographic LVH (ECG-LVH) was 4.3% [6], while another study in China reported the ECG-LVH prevalence among people with hypertension was 8.8% [7]. Robust evidence demonstrated that hypertension is the essential risk for developing LVH [2, 8, 9]. Although echocardiography is a gold standard for detecting LVH [10], the technology requires resources and specialists to complete the process. Whereas the Utrecht Health Project pointed out that routinely recording an electrocardiography (ECG) in unselected hypertension patients is considerable, the number needed to screen to prevent one death is lower than that in other widely accepted tests [11]. In Thailand, the Thai guideline for treating hypertension recommended that all Thai patients with hypertension should receive 12-lead ECG screening to detect ECG abnormalities [12].

Regarding the National Health Examination Survey (NHES) reports in Thailand, in 2003–2004 (NHES III), the hypertension prevalence was 23.3% in men and 20.9% in women, and increased to 26.7% in men and 24.2% in women in 2019–2020 (NHES VI) [13–16]. In 2018, the Thailand Diabetes Mellitus/Hypertension (DM/HT) study reported that the overall prevalence of blood pressure (BP) control among patients with hypertension receiving continuous care for at least 12 months was 66.6% at the latest visit [17]. Although small-scale study results are available for the specific population in Thailand [9, 18], the information on LVH among Thai patients with hypertension was not well characterized. We, therefore, aimed to identify the prevalence and factors associated with ECG-LVH among patients with hypertension in Thailand.

## Methods

### Study design and subjects

We utilized the secondary data of the Thailand DM/HT dataset, which conducted a serial cross-sectional study among Thai patients with hypertension in 2011–2015 and 2018, after permission from the National Health Security Office of Thailand [17, 19]. Briefly, the Thailand DM/HT study was a series of annual surveys in 2011–2015 and 2018 to evaluate the clinical outcomes of people with hypertension and type 2 diabetes (T2D) aged 20 years and older receiving continuous care for at least 12 months at clinics in hospitals nationwide [17]. The study employed a rigorous multistage sampling method proportional to the size to select a national and provincial sample of patients from 77 provinces nationwide [17]. The database consisted of two separate databases, one focusing on patients with hypertension and the other on patients with T2D. For the present study, we obtained the dataset focusing on the patients with hypertension, some of whom may have T2D comorbidity. The present study enrolled 226,420 people with hypertension between 2011 and 2018. Our objective was to determine the prevalence of ECG-LVH and associated factors; thus, participants without ECG data in the medical record would be excluded (*n* = 187,613). Therefore, 38,807 participants were eligible and included in the analysis (Table S1).

### Data collection

Regarding the Thailand DM/HT study, a well-trained registered nurse reviewed and retrieved the data using a standardized case report form (CRF) according to a standard protocol and sent them to the data management unit [17, 19]. The characteristics of study participants included sex, age, health insurance scheme, geographic region, hospital levels where patients receive hypertension care, and duration of hypertension. T2D comorbidity was defined as a history of T2D diagnosis or antihyperglycemic drug use or fasting plasma glucose (FPG) ≥126 mg/dL [20]. Tobacco use was defined as the smoking status presented in the medical records: never smoked, ex-smokers, and current smokers. In addition, height and weight in the latest visit were collected and calculated to body mass index (BMI). The BP information in the latest visit was also collected. Control BP was defined as systolic BP (SBP) < 140 mmHg and diastolic BP (DBP) < 90 mmHg [12]. The data on the use of antihypertensive medication was available only for the years 2014, 2015, and 2018. The medications used were categorized into single therapy, dual therapy, polytherapy, and no medication used. The medication patterns were classified into 12 categories: (1) angiotensin-converting enzyme inhibitors (ACEI) or angiotensin receptor blockers (ARB) only; (2) calcium channel blockers (CCB) only; (3) β-blockers (BB) only; (4) diuretics only; (5) ACEI/ARB + BB; (6) ACEI/ARB + CCB; (7) ACEI/ARB + diuretics; (8) CCB + BB; (9) CCB + diuretics; (10) BB + diuretics; (11) ACEI/ARB + BB + CCB; and (12) others. For participants who had the information on ECG in the medical records, the results of the ECG interpretation were retrieved and recorded in the CRF. In Thailand, medical practitioners often rely on voltage criteria for interpreting the ECG-LVH. The Sokolow-Lyon voltage (SV1 + RV5/6) > 35 mm [21] and the Cornell voltage criterion-based LVH are two such criteria commonly used. The latter is defined as R in aVL + SV3 ≥ 28 mm for men and S in V3 + R in aVL > 20 mm for wome n[22]. ECG-LVH was noted for the presence of LVH.

## Statistical analysis

All statistical analyses were performed using Stata ver. 17 (Stata Corp). The characteristics of study participants were analyzed using descriptive statistics. Categorial variables were presented as percentages, while continuous variables were presented as mean and standard deviation. The overall prevalence of ECG-LVH was calculated and presented as a percentage and 95% confidence interval (CI). Sex-specific prevalence of ECG-LVH was adjusted for five age categories (< 50, 50–59, 60–69, 70–79, and ≥ 80 years). The chi-square test was utilized to compare the distribution of ECG-LVH prevalence across the characteristics of study participants. Logistic regression analysis was employed to identify the factors associated with ECG-LVH prevalence. The multivariable analysis was performed to estimate the adjusted odds ratio (AOR) and 95% CI. Variables, including sex, age, health insurance scheme, geographic region, hospital level, T2D comorbidity, BMI, hypertension duration, smoking status, and control BP, were included in the final model. Additionally, the interaction was tested to explore whether sex modifies the association between age and ECG-LVH prevalence. The association between age and ECG-LVH was then identified among men and women. A two-sided *P*-value less than 0.05 was considered statistically significant.

## Sensitivity analysis

We performed a sensitivity analysis to account for patients with hypertension whose ECG records did not have. Marginal structural models (MSMs) were used to determine factors associated with ECG-LVH among study participants. We construct stabilized inverse probability attrition weight (IPAW) for this analysis. Logistic regression was utilized to model not having an ECG record conditional on sex, age, health scheme, geographic region, and hospital level to predict IPAW. Further, the logistic regression was also used to predict not having an ECG record to estimate the stabilized constant for IPAW. The stabilized constant divided by IPAW calculated the stabilized IPAW. The svyset with pw command was used to set the stabilized IPAW for MSMs. Then, a multivariable logistic regression model was used to determine factors associated with ECG-LVH and presented as AOR and 95% CI. We also took into account the utilization of antihypertensive medication, which was only accessible in the dataset for certain years (2014, 2015, and 2018). In our multivariable logistic regression analysis and MSMs, we incorporated the number and category of antihypertensive medications utilized. Ultimately, despite adjusting for potential confounders in the multivariable model in the primary analysis, there is still the possibility of residual confounding effects. As a result, we conducted a sensitivity analysis for unmeasured confounding using *E*-values estimated by the evalue package (Stata Corp) [23].

## Ethics statement

This study was reviewed and approved by the Institutional Review Board of the Royal Thai Army Medical Department (No. S055h/65_Exp), with a waiver for informed consent as secondary data were used. The study was conducted in compliance with various international guidelines, including the Declaration of Helsinki, the Belmont Report, the Council for International Organizations of Medical Sciences guidelines, and the Good Clinical Practice of the International Conference on Harmonization of Technical Requirements for Registration of Pharmaceuticals for Human Use.

## Results

### Characteristics of study participants

A total of 226,420 patients with hypertension participated in the Thailand DM/HT study in 2011–2015 and 2018. Of those, 38,807 individuals (17.1%) with ECG data recorded were included in the analysis. Table 1 presents the characteristics of study participants. The mean age was 64.8 ± 11.5 years, and 62.2% were women. The majority of study participants (72.1%) were under a universal health coverage scheme, and approximately one-third of participants (33.7%) resided in the central region. Mean SBP was 131.9 ± 15.7 mmHg while mean DBP was 75.3 ± 10.9 mmHg.

**Table 1:** Characteristics of study participants (*n* = 38,807)

Characteristic	Value
Sex	
Female	24,131 (62.2)
Male	14,676 (37.8)
Age (yr)	64.8 ± 11.5
20–49	3749 (9.7)
50–59	8969 (23.1)
60–69	12,137 (31.3)
70–79	9966 (25.7)
≥ 80	3986 (10.3)
Health insurance scheme	
Universal health coverage	27,965 (72.1)
Civil servant medical benefit	9129 (23.5)
Social security	1391 (3.6)
Other	322 (0.8)
Region	
Northeast	8001 (20.6)
North	10,932 (28.2)
Central	13,075 (33.7)
South	6799 (17.5)
Hospital level	
Community hospital	24,080 (62.1)
Provincial hospital	10,969 (28.3)
Regional hospital	3758 (9.7)
Type 2 diabetes comorbidity	
No	26,338 (67.9)
Yes	12,469 (32.1)
Smoking status	
Never	26,570 (68.5)
Ex-smoker	3934 (10.1)
Current smoker	6319 (16.3)
Missing	1984 (5.1)
Body mass index (kg/m^2^)	25.0 ± 4.6
< 30	32,133 (82.8)
≥ 30	4603 (11.9)
Missing	2071 (5.3)
Systolic blood pressure (mmHg)	131.9 ± 15.7
< 140	27,178 (70.1)
≥ 140	11,584 (29.9)
Diastolic blood pressure (mmHg)	75.3 ± 10.9
< 90	34,241 (88.2)
≥ 90	4500 (11.6)
No. of antihypertensive medication use (*n* = 17,963)^a^	
Poly therapy	3150 (17.5)
Dual therapy	7295 (40.6)
Single therapy	7146 (39.8)
No medication use	372 (2.1)
Category of antihypertensive medication use (*n* = 17,963)^a^	
ACEI/ARB only	2845 (15.8)
CCB only	2889 (16.1)
BB only	741 (4.1)
Diuretics only	602 (3.4)
ACEI/ARB + CCB	3043 (16.9)
ACEI/ARB + BB	1046 (5.8)
ACEI/ARB + diuretics	895 (5.0)
CCB + BB	975 (5.4)
CCB + diuretics	641 (3.7)
BB + diuretics	345 (1.9)
ACEI/ARB + BB + CCB	1363 (7.6)
Other	2578 (14.4)

Data are presented as number (%) or mean ± standard deviation. Percentages may not total 100 due to rounding

*ACEI* angiotensin-converting enzyme inhibitors, *ARB* angiotensin receptor blockers, *CCB* calcium channel blockers, *BB* β-blockers

^a^The data on the use of antihypertensive medication was available only for the years 2014, 2015, and 2018

### Prevalence of ECG-LVH among Thai patients with hypertension

**Fig. 1:**
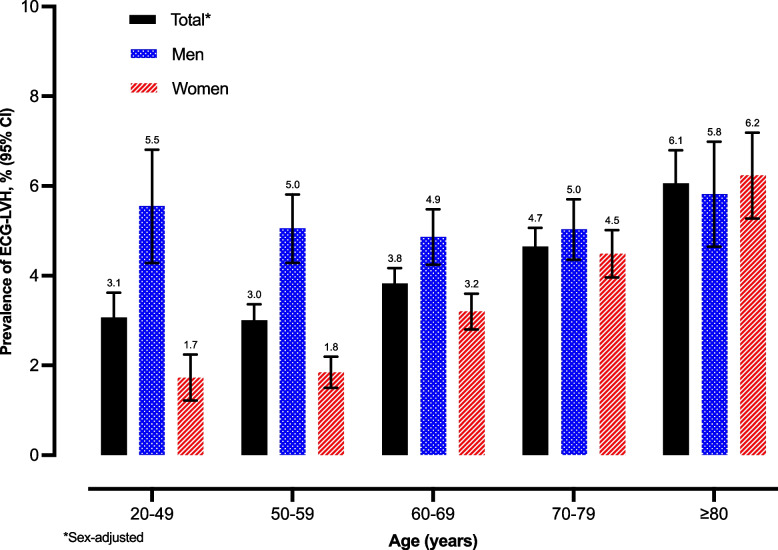
Age- and sex-specific prevalence of electrocardiographic left ventricular hypertrophy (ECG-LVH) among Thai patients with hypertension (2011–2018). CI, confidence interval

Overall, 1,557 study participants had ECG-LVH, with an estimated prevalence of 4.0% (95% CI, 3.8–4.2%). Age-adjusted ECG-LVH prevalence was 3.4% (95% CI, 3.1–3.6%) among women and 5.1% (95% CI, 4.7–5.4%) among men (*P* < 0.001). A higher ECG-LVH prevalence was observed regarding higher age (Fig. 1). Table 2 displays the distribution of ECG-LVH prevalence across the characteristics of participants. Notably, the findings indicate that patients with hypertension in the central, north, south, and northeast regions had ECG-LVH prevalence rates of 4.6, 4.3, 3.9, and 2.8%, respectively. Moreover, patients receiving hypertension care at regional hospitals exhibited higher ECG-LVH prevalence than those receiving care at provincial and community hospitals. Interestingly, the data also revealed that patients with longer hypertension duration, particularly those with a duration exceeding 20 years, had higher ECG-LVH prevalence.

**Table 2:** Distribution of electrocardiographic left ventricular hypertrophy prevalence across characteristics of participants

Characteristic	Left ventricular hypertrophy		*P*-value
	Yes	No	
Overall	1,557 (4.0)	37,250 (96.0)	
Sex			< 0.001
Female	808 (3.4)	23,323 (96.7)	
Male	749 (5.1)	13,927 (94.9)	
Age (yr)			< 0.001
20–49	113 (3.0)	3636 (97.0)	
50–59	267 (3.0)	8702 (97.0)	
60–69	466 (3.8)	11,671 (96.2)	
70–79	469 (4.7)	9497 (95.3)	
≥ 80	242 (6.1)	3744 (93.9)	
Health insurance scheme			0.381
Universal health coverage	1151 (4.1)	26,814 (95.9)	
Civil servant medical benefit	338 (3.7)	8791 (96.3)	
Social security	55 (4.0)	1336 (96.1)	
Other	13 (4.0)	309 (96.0)	
Region			< 0.001
Northeast	224 (2.8)	7777 (97.2)	
North	470 (4.3)	10,462 (95.7)	
Central	601 (4.6)	12,474 (95.4)	
South	262 (3.9)	6537 (96.2)	
Hospital level			< 0.001
Community hospital	792 (3.3)	23,288 (96.7)	
Provincial hospital	513 (4.7)	10,456 (95.3)	
Regional hospital	252 (6.7)	3506 (93.3)	
Type 2 diabetes comorbidity			0.217
No	1079 (4.1)	25,259 (95.9)	
Yes	478 (3.8)	11,991 (96.2)	
Smoking status			< 0.001
Never	975 (3.7)	25,595 (96.3)	
Ex-smoker	210 (5.3)	3724 (94.7)	
Current smoker	294 (4.7)	6025 (95.4)	
Hypertension duration (yr)			< 0.001
1–9	1037 (3.9)	25,892 (96.2)	
10–19	268 (4.9)	5243 (95.1)	
≥ 20	25 (7.5)	308 (92.5)	
Body mass index (kg/m^2^)			0.003
< 30	1284 (4.0)	30,849 (96.0)	
≥ 30	143 (3.1)	4460 (96.9)	
Systolic blood pressure (mmHg)			< 0.001
< 140	971 (3.6)	26,207 (96.4)	
≥ 140	584 (5.0)	11,000 (95.0)	
Diastolic blood pressure (mmHg)			0.002
< 90	1336 (3.9)	32,905 (96.1)	
≥ 90	218 (4.8)	4282 (95.2)	

Data are presented as number (%)

### Factors associated with ECG-LVH among Thai patients with hypertension

Table 3 presents univariable and multivariable analyses for factors associated with ECG-LVH. Factors associated with ECG-LVH included being men (AOR, 1.49; 95% CI, 1.31–1.69), individuals aged 60 to 69 years (AOR, 1.31; 95% CI, 1.02–1.68), those aged 70 to 79 years (AOR, 1.56; 95% CI, 1.20–2.02), and those ≥80 years (AOR, 2.10; 95% CI, 1.58–2.78) compared to individuals aged less than 50 years. The effect modification on the association between age and ECG-LVH was observed (Table 4). Patients with hypertension residing in the central region tended to have higher ECG-LVH than those in the northeast region (AOR, 1.62; 95% CI, 1.34–1.95). Study participants receiving hypertension treatment in clinics at regional and provincial hospitals tended to have higher ECG-LVH prevalence (regional hospital: AOR, 2.41; 95% CI, 2.01–2.89; provincial hospital: AOR, 1.46; 95% CI, 1.27–1.67) compared to those visiting community hospitals. Individuals with longer duration of hypertension tended to have more ECG-LVH prevalence: 10 to 19 years (AOR, 1.19; 95% CI, 1.03–139) and ≥ 20 years (AOR, 1.88; 95% CI, 1.22–2.91) compared to those with hypertension duration of less than 10 years. The modifiable factors associated with ECG-LVH included tobacco use and high BP. Current smokers tended to have ECG-LVH greater than those who never smoked (AOR, 1.26; 95% CI, 1.09–1.46). Meanwhile, participants with SBP ≥140 mmHg and DBP < 90 mmHg (AOR, 1.36; 95% CI, 1.18–1.56) and SBP ≥140 mmHg and DBP ≥90 mmHg (AOR, 1.58; 95% CI, 1.30–1.92) have a higher prevalence of ECG-LVH compared to those with SBP < 140 mmHg and DBP < 90 mmHg.

**Table 3:** Univariable and multivariable analysis for factors associated with electrocardiographic left ventricular hypertrophy

Characteristic	Univariable analysis		Multivariable analysis^a^	
	Crude OR (95% CI)	*P*-value	AOR (95% CI)	*P*-value
Sex				
Female	Reference	–	Reference	–
Male	1.55 (1.40–1.72)	< 0.001	1.49 (1.31–1.69)	< 0.001
Age (yr)				
20–49	Reference	–	Reference	–
50–59	0.99 (0.79–1.23)	0.911	0.95 (0.73–1.24)	0.696
60–69	1.28 (1.04–1.58)	0.019	1.31 (1.02–1.68)	0.038
70–79	1.59 (1.29–1.96)	< 0.001	1.56 (1.20–2.02)	0.001
≥ 80	2.08 (1.66–2.61)	< 0.001	2.10 (1.58–2.78)	< 0.001
Health insurance scheme				
Universal health coverage	Reference	–	Reference	–
Civil servant medical benefit	0.90 (0.79–1.01)	0.081	0.73 (0.63–0.84)	< 0.001
Social security	0.96 (0.73–1.26)	0.767	1.07 (0.77–1.46)	0.697
Other	0.98 (0.56–1.71)	0.944	1.02 (0.52–2.01)	0.956
Region				
Northeast	Reference	–	Reference	–
North	1.56 (1.33–1.83)	< 0.001	1.58 (1.30–1.91)	< 0.001
Central	1.67 (1.43–1.96)	< 0.001	1.62 (1.34–1.95)	< 0.001
South	1.39 (1.16–1.67)	< 0.001	1.39 (1.12–1.72)	0.003
Hospital level				
Community hospital	Reference	–	Reference	–
Provincial hospital	1.44 (1.29–1.62)	< 0.001	1.46 (1.27–1.67)	< 0.001
Regional hospital	2.11 (1.83–2.45)	< 0.001	2.41 (2.01–2.89)	< 0.001
Type 2 diabetes comorbidity				
No	Reference	–	Reference	–
Yes	0.93 (0.84–1.04)	0.217	0.89 (0.79–1.02)	0.085
Smoking status				
Never	Reference	–	Reference	–
Ex-smoker	1.48 (1.27–1.73)	< 0.001	1.18 (0.98–1.41)	0.086
Current smoker	1.28 (1.12–1.46)	< 0.001	1.26 (1.09–1.46)	0.002
Hypertension duration (yr)				
1–9	Reference	–	Reference	–
10–19	1.28 (1.11–1.46)	0.001	1.19 (1.03–1.39)	0.019
≥ 20	2.03 (1.34–3.06)	0.001	1.88 (1.22–2.91)	0.005
Body mass index (kg/m^2^)				
< 30	Reference	–	Reference	–
≥ 30	0.77 (0.65–0.92)	0.004	0.88 (0.72–1.08)	0.216
Blood pressure (mmHg)				
SBP < 140 and DBP < 90	Reference	–	Reference	–
SBP < 140 and DBP ≥90	1.09 (0.82–1.44)	0.569	1.00 (0.69–1.44)	0.997
SBP ≥140 and DBP < 90	1.42 (1.26–1.59)	< 0.001	1.36 (1.18–1.56)	< 0.001
SBP ≥140 and DBP ≥90	1.51 (1.27–1.79)	< 0.001	1.58 (1.30–1.92)	< 0.001

*OR* odds ratio, *AOR* adjusted odds ratio, *CI* confidence interval

^a^Adjusted for sex, age, health insurance scheme, geographic region, hospital level, type 2 diabetes comorbidity, body mass index, hypertension duration, smoking status, and control blood pressure

**Table 4:** Associated between age and electrocardiographic left ventricular hypertrophy in men and women

Age (yr)	Men^a^		Women^a^	
	AOR (95% CI)	*P*-value	AOR (95% CI)	*P*-value
20–49	Reference	–	Reference	–
50–59	0.87 (0.61–1.22)	0.410	1.04 (0.68–1.57)	0.871
60–69^*^	0.92 (0.66–1.28)	0.606	1.88 (1.27–2.79)	0.002
70–79^*^	0.99 (0.70–1.40)	0.966	2.44 (1.64–3.63)	< 0.001
≥80^*^	1.02 (0.68–1.52)	0.922	3.89 (2.56–5.91)	< 0.001

*AOR* adjusted odds ratio, *CI* confidence interval

^a^Adjusted for age, health insurance scheme, geographic region, hospital level, type 2 diabetes comorbidity, body mass index, hypertension duration, smoking status, and control blood pressure

^*^P for interaction < 0.05

The study used MSMs for sensitivity analysis to account for patients with hypertension who did not have an ECG record. The results showed that the factors associated with ECG-LVH among the study participants followed a pattern similar to that of the primary analysis (Tables S2, S3). Table S4 presented the ECG-LVH prevalence across different antihypertensive medication categories. Additionally, by including the number of medications used (Tables S5, S6) and the category of antihypertensive medication use (Tables S7, S8) in the multivariable analysis and the MSMs, the study found that the factors associated with ECG-LVH remained consistent with the primary analysis. The *E*-value for the odds ratio to identify the association between the unmeasured confounder and the factors associated with ECG-LVH is presented in Table S9.

## Discussion

The present study estimated the ECG-LVH prevalence among Thai patients with hypertension who received continuous care, accounting for 4.0%. ECG-LVH is more prevalent in men than women; older patients with hypertension are more likely to have it than younger patients. Modifiable risk factors associated with ECG-LVH include current tobacco use and uncontrolled hypertension.

A few studies in a specific population in Thailand estimated the prevalence of ECG-LVH. For instance, in 2000, Sriratanasathavorn et al. [18] reported that the ECG-LVH prevalence among private company officers aged 30 years and older in Bangkok was 13.0% [18]. Meanwhile, in 2020, a related study among adults aged 20 years and older in a Thai rural community in the central region estimated that the ECG-LVH prevalence was 6.6% [9]. A large-scale cohort study conducted in the United States involving an average follow-up period of 7 years revealed that the incidence of new-onset ECG-LVH was 3.0 and 4.3% in men and women with hypertension, respectively [6]. Another cross-sectional study conducted in the Chinese population reported that the prevalence of ECG-LVH in individuals with hypertension was 8.8% [7]. Our present study, however, observed a relatively low prevalence of 4.0% among participants. Notably, only 17.1% of patients with hypertension had ECG recorded. Therefore, the ECG-LVH prevalence in the present study may be underestimated.

Our study found that men have a higher incidence of ECG-LVH compared to women, which is consistent with related studies in Thailand [9, 18]. However, another study showed that women are at a higher risk of developing ECG-LVH [24]. While some studies suggest that men tend to have LVH, it was found that LVH has a greater impact on the survival of women than men [25]. This means that among individuals with LVH, women have a higher risk of cardiac mortality, with a risk that is three to five times higher than that of men [25]. Therefore, we recommend that all patients with hypertension undergo a 12-lead ECG screening. If LVH is detected, appropriate management should be initiated to mitigate the risk of CVD complications such as IHD, stroke, and sudden cardiac death [3, 4, 26].

Robust evidence demonstrated that advanced age was associated with greater left ventricular (LV) wall thickness and LV mass [27–29]. Similarly, our results indicated the association between higher age and ECG-LVH with a dose-response relationship, particularly in patients over 60 years old. This older-aged population, especially those who develop LVH, tended to have aging-associated diseases, including CVD [3, 30]. However, this pattern was not observed in male participants. We found that the prevalence of ECG-LVH was consistently high in men, regardless of age, and was relatively higher compared to women in the same age group. This can be attributed to the effect of sex on the association between age and ECG-LVH. Therefore, it is important to closely monitor LVH in older patients with hypertension, and to emphasize ECG screening for men with hypertension of all ages.

According to an extensive study, the distribution of ECG-LVH among Thai patients with hypertension was illustrated across the country. The study found that the prevalence of ECG-LVH among participants in the central region tended to be higher than in other regions. In 2018, a previous study by Sakboonyarat and Rangsin [31] also reported a higher prevalence of IHD in the central region compared to other regions [31]. Since LVH is a predictor of CVD, including IHD, our results suggest that emphasis should be given to early detection of ECG-LVH. If ECG-LVH is detected, prompt management such as routine monitoring, effective treatment [32], and modification of lifestyle risk factors should be performed [33].

Our findings indicate that smoking is strongly associated with a 26-percentage point prevalence of ECG-LVH compared to non-smokers. Robust evidence demonstrated that smoking affects the cardiac structure, including increased LV mass index and LVH [34–36]. Additionally, tobacco constituents, particularly nicotine, can contribute to ventricular tachycardia (VT) and ventricular fibrillation (VF) via sympathetic stimulation [37]. The life-threatening complications in LVH include VT/VF and sudden cardiac death [5, 37]. Consequently, we strongly suggest that hypertensive patients who currently smoke and have detected LVH should quit smoking, while those without LVH should be encouraged to stop smoking.

Long-term high BP strains the LV, resulting in LVH [2, 8]. The present study revealed that participants who had been suffering from hypertension for longer periods of time were more likely to have ECG-LVH than those who had shorter durations of hypertension. Moreover, our results demonstrated that participants with uncontrolled hypertension tended to have ECG-LVH. Compared to individuals with control BP, those with only SBP ≥140 mmHg have a 36 percentage point higher ECG-LVH prevalence. Furthermore, those with SBP ≥140 mmHg and DBP ≥90 mmHg have a 58 percentage point higher ECG-LVH prevalence. Similarly, one related study in the Thai rural community demonstrated that people with a hypertension crisis (BP ≥180/110 mmHg) have ECG-LVH 7.2 times greater than those with BP < 180/110 mmHg [9]. Our findings emphasized the importance of high BP as a modifiable risk factor for ECG-LVH. BP control, both SBP and DBP, should be facilitated among hypertension patients to mitigate the risk of LVH and CVD sequel later [38, 39].

The extant literature suggests that LVH is widespread among individuals with T2D [40]. However, our study found that only 3.8% of T2D patients had LVH detected through ECG, notably lower than the previously reported estimates of LVH prevalence in T2D, ranging from 32 to 71% [40–43]. This discrepancy could be attributed to different diagnostic tools across studies, with most studies using echocardiography to detect LVH. Furthermore, the low sensitivity of ECG in detecting LVH could also be a contributing factor. Notably, a recent diagnostic study based on data from the Framingham Heart Study reported an overall sensitivity of ECG-LVH of 6.9% and specificity of 98.8% compared to echocardiography as the reference standard [44].

The present study has some limitations. First, the study utilized secondary data from the Thailand DM/HT study. The information on ECG data was reviewed and retrieved from medical records, and only 17.1% of participants had ECG data. However, we found that the distribution of characteristics between study participants and those without ECG was comparable (Table S1). Due to having a slightly higher proportion of men and older participants with ECG compared with those without ECG, we performed a sensitivity analysis to estimate the ECG-LVH prevalence standardized to the age and sex distribution of all patients both with and without ECG data (*n* = 226,420). The standardized ECG-LVH prevalence was 4.1%. Nevertheless, if the ECG screening was not performed randomly, the estimated ECG-LVH prevalence in the present study would be interpreted carefully within the participants included in the analysis. Despite these limitations, the NHES VI revealed that the sex distribution of Thai adults with HT receiving treatment was 61.6% for women and 38.4% for men [16, 17]. This is compatible with the sex distribution of participants in the present study. Therefore, the results may be feasible to represent the situation among patients with hypertension receiving continuous care in Thailand. Additionally, to account for those patients whose ECG records did not have, we performed a sensitivity analysis using the MSMs. The results demonstrated that factors associated with ECG-LVH obtained from the MSMs were compatible with the primary analysis and did not change the conclusion.

Second, although a registered nurse conducted the medical record review, the ECG-LVH cases were defined by reading the ECG interpretation in the medical records, which may have caused misclassification. Despite our best efforts to provide a comprehensive analysis through multiple stages of sensitivity analysis, certain factors such as infrequent ECG measurements and potential inaccuracies in the interpretation of LVH may still impact the reliability of our findings at a national level. Therefore, it is recommended that future research endeavors attempt to address these gaps in order to improve the representativeness of national studies.

Third, the present study employed a cross-sectional design, making causal inference difficult. Fourth, the information on antihypertensive medication uses was available only in 2014, 2015, and 2018, but sensitivity analysis showed no change in the conclusion from the primary analysis. Regarding the secondary database utilization, other lifestyle factors, including alcohol use, physical activity, and medication adherence, were not included in the final model. Therefore, residual confounding may exist. The evidence for causality from the *E*-values (Table S9) looks relatively strong because substantial unmeasured confounding would be needed to reduce the observed association between factors and ECG-LVH. The present study had considerable strengths. To our knowledge, it is an extensive and the most recent study conducted to illustrate and provide insight into ECG-LVH among Thai hypertension patients.

## Conclusions

The present study demonstrated the prevalence of ECG-LVH among Thai patients with hypertension who underwent ECG recording and identified high-risk groups that were more likely to have ECG-LVH. The study highlights the importance of targeted interventions, especially among high-risk groups such as older individuals, men, and current smokers, to address the modifiable factors linked to ECG-LVH.

### Supplementary Information

**Additional file 1: Table S1.** Characteristics of patients with hypertension between inclusion and exclusion groups. **Table S2.** Multivariable analysis for factors associated with ECG-LVH using marginal structural models (model M1). **Table S3.** Associated between age and ECG-LVH in men and women using marginal structural models (model M2). **Table S4.** Univariable for the association between antihypertensive medications use and ECG-LVH (unadjusted model). **Table S5.** Multivariable analysis for factors associated with ECG-LVH (models M3 and M4); added number of antihypertensive medications use. **Table S6.** Associated between age and ECG-LVH in men and women (models M5 and M6); added number of antihypertensive medications use. **Table S7.** Multivariable analysis for factors associated with ECG-LVH (models M7 and M8); added antihypertensive medications use categories. **Table S8.** Associated between age and ECG-LVH in men and women (models M9 and M10); added antihypertensive medications use categories. **Table S9.** Sensitivity analysis for unmeasured confounding using *E*-value for odds ratio.

## Data Availability

Data cannot be shared publicly because the data set contains identifying information; additionally, the data belong to the Thailand Diabetes Mellitus/Hypertension study of the Medical Research Network of the Consortium of Thai Medical Schools (MedResNet). Thus, ethical restrictions exist on the data set. Data are available from the Thai National Health Security Office (https://dmht.thaimedresnet.org/) for researchers who meet the criteria for access to confidential data.

## Abbreviations

ACEI: Angiotensin-converting enzyme inhibitors
AOR: Adjusted odds ratio
ARB: Angiotensin receptor blockers
BB: β-blockers
BMI: Body mass index
BP: Blood pressure
CCB: Calcium channel blockers
CI: Confidence interval
CRF: Case report form
CVD: Cardiovascular disease
DBP: Diastolic blood pressure
DM/HT: Diabetes Mellitus/Hypertension
ECG: Electrocardiography
ECG-LVH: Electrocardiographic left ventricular hypertrophy
FPG: Fasting plasma glucose
IHD: Ischemic heart disease
IPAW: Inverse probability attrition weight
LV: Left ventricular
LVH: Left ventricular hypertrophy
MSM: Marginal structural model
NHES: National health examination survey
OR: Odds ratio
SBP: Systolic blood pressure
T2D: Type 2 diabetes
VF: Ventricular fibrillation
VT: Ventricular tachycardia
