# Supplementary material for: Factors associated with electrocardiographic left ventricular hypertrophy among patients with hypertension in Thailand
Source: Clin Hypertens. 2024 Apr 1;30:8. doi: 10.1186/s40885-024-00267-8 (PMC10983697; doi:10.1186/s40885-024-00267-8)
Supplement: Supplementary file 1 — Additional file 1: Table S1. Characteristics of patients with hypertension between inclusion and exclusion groups. Table S2. Multivariable analysis for factors associated with ECG-LVH using marginal structural models (model M1). Table S3. Associated between age and ECG-LVH in men and women using marginal structural models (model M2). Table S4. Univariable for the association between antihypertensive medications use and ECG-LVH (unadjusted model). Table S5. Multivariable analysis for factors associated with ECG-LVH (models M3 and M4); added number of antihypertensive medications use. Table S6. Associated between age and ECG-LVH in men and women (models M5 and M6); added number of antihypertensive medications use. Table S7. Multivariable analysis for factors associated with ECG-LVH (models M7 and M8); added antihypertensive medications use categories. Table S8. Associated between age and ECG-LVH in men and women (models M9 and M10); added antihypertensive medications use categories. Table S9. Sensitivity analysis for unmeasured confounding using E-value for odds ratio. [file 40885_2024_267_MOESM1_ESM.docx]

**Appendix**

**Factors associated with electrocardiographic left ventricular hypertrophy among patients with hypertension in Thailand**

**Boonsub Sakboonyarat^1^, Jaturon Poovieng^2^, Ram Rangsin^1^*

^1^Department of Military and Community Medicine, Phramongkutklao College of Medicine, Bangkok 10400, Thailand

^2^Pulmonary and Critical Care Division, Department of Medicine, Phramongkutklao College of Medicine, Bangkok 10400, Thailand

***Corresponding author**

Boonsub Sakboonyarat, MD, MPH

Department of Military and Community Medicine, Phramongkutklao College of Medicine, Bangkok 10400, Thailand

E-mail: boonsub1991@pcm.ac.th

Tel: +668-5954-5955

**Table of Contents**

| Table **S1** Characteristics of patients with hypertension between inclusion and exclusion groups |  | Page 3 |
| --- | --- | --- |
| Table **S2** Multivariable analysis for factors associated with ECG-LVH using marginal structural models (Model M1) |  | Page 4 |
| Table **S3** Associated between age and ECG-LVH in men and women using marginal structural models (Model M2) |  | Page 5 |
| Table **S4** Univariable for the association between antihypertensive medications use  And ECG-LVH (unadjusted model) |  | Page 6 |
| Table **S5** Multivariable analysis for factors associated with ECG-LVH (Model M3 and Model M4); added number of antihypertensive medications use |  | Page 7 |
| Table **S6** Associated between age and ECG-LVH in men and women (Model M5 and Model M6); added number of antihypertensive medications use |  | Page 8 |
| Table **S7** Multivariable analysis for factors associated with ECG-LVH (Model M7 and Model M8); added antihypertensive medications use categories |  | Page 9 |
| Table **S8** Associated between age and ECG-LVH in men and women (Model M9 and Model M10); added antihypertensive medications use categories |  | Page 10 |
| Table **S9.** Sensitivity analysis for unmeasured confounding using E-value for odds ratio. |  | Page 11 |

**Table S1.** Characteristics of patients with hypertension between inclusion and exclusion groups

| **Characterisitc** | **Having information on12-lead ECG** | **No information on 12-lead ECG^a)^** |
| --- | --- | --- |
|  | **N (%)** | **N (%)** |
| **Distribution** | **38,807 (17.1%)** | **187,613 (82.9%)** |
| **Sex** |  |  |
| Women | 24131 (62.2) | 119128 (63.5) |
| Men | 14676 (37.8) | 68485 (36.5) |
| **Age, years** |  |  |
| 20-49 | 3749 (9.7) | 22907 (12.2) |
| 50-59 | 8969 (23.1) | 49717 (26.5) |
| 60-69 | 12137 (31.3) | 58428 (31.1) |
| 70-79 | 9966 (25.7) | 41939 (22.4) |
| ≥80 | 3986 (10.3) | 14622 (7.8) |
| mean (SD) | 64.8 (11.5) | 63.1 (11.4) |
| **Health insurance scheme** |  |  |
| Universal health coverage | 27965 (72.1) | 141046 (75.2) |
| Civil servant medical benefit | 9129 (23.5) | 37220 (19.8) |
| Social security | 1391 (3.6) | 7809 (4.2) |
| Others | 322 (0.8) | 1538 (0.8) |
| **Regions** |  |  |
| Northeast | 8001 (20.6) | 50055 (26.7) |
| North | 10932 (28.2) | 47346 (25.2) |
| Central | 13075 (33.7) | 62681 (33.4) |
| South | 6799 (17.5) | 27531 (14.7) |
| **Hospital levels** |  |  |
| Community hospital | 24080 (62.1) | 128283 (68.4) |
| Provincial hospotal | 10969 (28.3) | 39325 (21.0) |
| Regional hospital | 3758 (9.7) | 20005 (10.7) |

^a)^Excluded from the analysis in the present study.

**Table S2.** Multivariable analysis for factors associated with ECG-LVH using marginal structural models

| **Factors** | **Model M1**^a)^ | |
| --- | --- | --- |
|  | **Adjusted OR (95% CI)** | ***p*- value** |
| **Sex** |  |  |
| Women | Ref. |  |
| Men | 1.54 (1.35-1.76) | <0.001 |
| **Age, years** |  |  |
| 20-49 | Ref. |  |
| 50-59 | 0.92 (0.70-1.20) | 0.535 |
| 60-69 | 1.27 (0.98-1.65) | 0.076 |
| 70-79 | 1.50 (1.15-1.96) | 0.003 |
| ≥80 | 2.07 (1.55-2.76) | <0.001 |
| **Health insurance scheme** |  |  |
| Universal health coverage | Ref. |  |
| Civil servant medical benefit | 0.71 (0.61-0.83) | <0.001 |
| Social security | 1.06 (0.76-1.47) | 0.726 |
| Others | 0.86 (0.43-1.74) | 0.681 |
| **Regions** |  |  |
| Northeast | Ref. |  |
| North | 1.53 (1.25-1.86) | <0.001 |
| Central | 1.65 (1.36-2.00) | <0.001 |
| South | 1.38 (1.11-1.72) | 0.004 |
| **Hospital levels** |  |  |
| Community hospital | Ref. |  |
| Provincial hospotal | 1.50 (1.30-1.72) | <0.001 |
| Regional hospital | 2.54 (2.10-3.08) | <0.001 |
| **Type 2 diabetes comorbidity** |  |  |
| No | Ref. |  |
| Yes | 0.91 (0.80-1.04) | 0.154 |
| **Smoking status** |  |  |
| Never | Ref. |  |
| Ex-smoker | 1.20 (0.99-1.45) | 0.065 |
| Current smoker | 1.26 (1.08-1.47) | 0.003 |
| **Hypertension duration, years** |  |  |
| 1-9 | Ref. |  |
| 10-19 | 1.18 (1.01-1.37) | 0.038 |
| ≥20 | 1.60 (1.03-2.49) | 0.036 |
| **Body mass index, kg/m^2^** |  |  |
| <30 | Ref. |  |
| ≥30 | 0.86 (0.7-1.06) | 0.152 |
| **Blood pressure, mmHg** |  |  |
| SBP <140 and DBP < 90 | Ref. |  |
| SBP <140 and DBP ≥ 90 | 1.05 (0.72-1.53) | 0.802 |
| SBP ≥140 and DBP < 90 | 1.39 (1.21-1.61) | <0.001 |
| SBP ≥140 and DBP ≥ 90 | 1.60 (1.31-1.97) | <0.001 |

^a)^**Model M1:** Adjusting for sex, age, health insurance scheme, geographic region, hospital level, T2D comorbidity, BMI, HTN duration, smoking status, and control BP.

ECG-LVH: Electrocardiographic left ventricular hypertrophy, OR: odds ratio, CI: confidence interval

**Table S3.** Associated between age and ECG-LVH in men and women (using marginal structural models)

| **Model M2** | **Men**^a)^ | | **Women**^a)^ | |
| --- | --- | --- | --- | --- |
|  | **Adjusted OR (95% CI)** | ***p* - value** | **Adjusted OR (95% CI)** | ***p* - value** |
| **Age, years** |  |  |  |  |
| 20-49 | Ref. |  | Ref. |  |
| 50-59 | 0.85 (0.60-1.20) | 0.351 | 0.99 (0.64-1.52) | 0.95 |
| 60-69^†^ | 0.89 (0.63-1.25) | 0.497 | 1.80 (1.18-2.72) | 0.006 |
| 70-79^†^ | 0.96 (0.68-1.36) | 0.828 | 2.31 (1.51-3.50) | <0.001 |
| ≥80^†^ | 0.97 (0.65-1.46) | 0.891 | 3.82 (2.47-5.93) | <0.001 |

^a)^**Model M2:** Adjusting for age, health insurance scheme, geographic region, hospital level, T2D comorbidity, BMI, HTN duration, smoking status, and control BP.

ECG-LVH: Electrocardiographic left ventricular hypertrophy, OR: odds ratio, CI: confidence interval

^†^*P* for interaction <0.05

Table **S4** Univariable analysis for the association between antihypertensive medications use

and ECG-LVH (unadjusted model)

| **Characterisitcs** | **ECG-LVH** | | **Univariable analysis** | |
| --- | --- | --- | --- | --- |
|  | **Yes, n (%)** | **No, n (%)** | **Crude OR (95% CI)** | ***p* - value** |
| **Number of anti-hypertensive medication use** | | |  |  |
| Poly therapy | 206 (6.5) | 2944 (93.5) | Ref. |  |
| Dual therapy | 319 (4.4) | 6976 (95.6) | 0.65 (0.55-0.78) | <0.001 |
| Single therapy | 251 (3.5) | 6895 (96.5) | 0.52 (0.43-0.63) | <0.001 |
| No medication use | 20 (5.4) | 352 (94.6) | 0.81 (0.51-1.30) | 0.387 |
| **Category of anti-hypertensive medication use** | | |  |  |
| ACEI/ARB only | 101 (3.6) | 2744 (96.4) | Ref. |  |
| CCB only | 91 (3.2) | 2798 (96.8) | 0.88 (0.66-1.18) | 0.400 |
| 𝛽-blockers only | 26 (3.5) | 715 (96.5) | 0.99 (0.64-1.53) | 0.957 |
| Diuretics only | 29 (4.8) | 573 (95.2) | 1.38 (0.90-2.10) | 0.14 |
| ACEI/ARB + CCB | 82 (2.7) | 2961 (97.3) | 0.75 (0.56-1.01) | 0.060 |
| ACEI/ARB + 𝛽-blockers | 63 (6.0) | 983 (94.0) | 1.74 (1.26-2.41) | 0.001 |
| ACEI/ARB + Diuretics | 61 (6.8) | 834 (93.2) | 1.99 (1.43-2.76) | <0.001 |
| CCB + 𝛽-blockers | 49 (5.0) | 926 (95.0) | 1.44 (1.01-2.04) | 0.042 |
| CCB + Diuretics | 29 (4.5) | 612 (95.5) | 1.29 (0.84-196) | 0.241 |
| 𝛽-blockers + Diuretics | 14 (4.1) | 331 (95.9) | 1.15 (0.65-2.03) | 0.633 |
| ACEI/ARB + 𝛽-blockers + CCB | 85 (6.2) | 1278 (93.8) | 1.81 (1.34-2.43) | <0.001 |
| Others | 166 (6.4) | 2412 (93.6) | 1.87 (1.45-2.41) | <0.001 |

ECG-LVH: Electrocardiographic left ventricular hypertrophy, OR: odds ratio, CI: confidence interval

The variables of antihypertensive medication were available on the dataset in 2014, 2015, and 2018; thus, the total N would be reduced from 38,807 (primary analysis) to 17,963.

**Table S5.** Multivariable analysis for factors associated with ECG-LVH (Model M3 and Model M4); added number of antihypertensive medications use

| **Factors** | **Model M3** ^a)^ | | **Model M4** ^b)^ | |
| --- | --- | --- | --- | --- |
|  | **AOR (95% CI)** | ***p* - value** | **AOR (95% CI)** | ***p* - value** |
| **Sex** |  |  |  |  |
| Women | Ref. |  | Ref. |  |
| Men | 1.32 (1.11-1.56) | 0.002 | 1.36 (1.14-1.62) | 0.001 |
| **Age, years** |  |  |  |  |
| 20-49 | Ref. |  | Ref. |  |
| 50-59 | 0.92 (0.66-1.28) | 0.619 | 0.89 (0.64-1.26) | 0.524 |
| 60-69 | 1.24 (0.90-1.71) | 0.18 | 1.27 (0.91-1.75) | 0.155 |
| 70-79 | 1.42 (1.03-1.97) | 0.034 | 1.41 (1.01-1.96) | 0.042 |
| ≥80 | 1.97 (1.39-2.79) | <0.001 | 2.03 (1.43-2.89) | <0.001 |
| **Health coverage scheme** |  |  |  |  |
| Universal health coverage | Ref. |  | Ref. |  |
| Civil servant medical benefit | 0.72 (0.60-0.87) | 0.001 | 0.71 (0.58-0.86) | 0.001 |
| Social security | 1.24 (0.84-1.82) | 0.276 | 1.25 (0.85-1.83) | 0.253 |
| Others | 0.86 (0.35-2.14) | 0.753 | 0.72 (0.28-1.88) | 0.503 |
| **Regions** |  |  |  |  |
| Northeast | Ref. |  | Ref. |  |
| North | 1.65 (1.29-2.12) | <0.001 | 1.62 (1.25-2.09) | <0.001 |
| Central | 1.46 (1.14-1.86) | 0.003 | 1.46 (1.13-1.88) | 0.003 |
| South | 1.48 (1.12-1.95) | 0.005 | 1.46 (1.10-1.94) | 0.009 |
| **Hospital levels** |  |  |  |  |
| Community hospital | Ref. |  | Ref. |  |
| Provincial hospotal | 1.54 (1.30-1.83) | <0.001 | 1.55 (1.30-1.85) | <0.001 |
| Regional hospital | 2.48 (1.94-3.17) | <0.001 | 2.80 (2.17-3.63) | <0.001 |
| **Type 2 diabetes comorbidity** |  |  |  | <0.001 |
| No | Ref. |  | Ref. |  |
| Yes | 1.07 (0.9-1.26) | 0.456 | 1.14 (0.95-1.36) | 0.154 |
| **Smoking status** |  |  |  |  |
| Never | Ref. |  | Ref. |  |
| Ex-smoker | 1.14 (0.91-1.42) | 0.248 | 1.16 (0.92-1.46) | 0.224 |
| Current smoker | 1.50 (1.08-2.09) | 0.017 | 1.49 (1.06-2.11) | 0.023 |
| **Hypertension duration, years** |  |  |  |  |
| 1-9 | Ref. |  | Ref. |  |
| 10-19 | 1.13 (0.94-1.36) | 0.181 | 1.13 (0.93-1.37) | 0.218 |
| ≥20 | 1.76 (1.00-3.10) | 0.049 | 1.52 (0.85-2.71) | 0.158 |
| **Body mass index, kg/m^2^** |  |  |  |  |
| <30 | Ref. |  | Ref. |  |
| ≥30 | 0.85 (0.66-1.09) | 0.203 | 0.87 (0.67-1.13) | 0.305 |
| **Blood pressure, mmHg** |  |  |  |  |
| SBP <140 and DBP < 90 | Ref. |  | Ref. |  |
| SBP <140 and DBP ≥ 90 | 1.02 (0.64-1.62) | 0.934 | 1.03 (0.63-1.68) | 0.905 |
| SBP ≥140 and DBP < 90 | 1.27 (1.07-1.51) | 0.007 | 1.27 (1.06-1.52) | 0.010 |
| SBP ≥140 and DBP ≥ 90 | 1.53 (1.20-1.96) | 0.001 | 1.56 (1.21-2.02) | 0.001 |
| **Number of anti-hypertensive medication use** | |  |  |  |
| Poly therapy | Ref. |  | Ref. |  |
| Dual therapy | 0.70 (0.58-0.84) | <0.001 | 0.71 (0.58-0.87) | 0.001 |
| Single therapy | 0.58 (0.47-0.71) | <0.001 | 0.58 (0.47-0.72) | <0.001 |
| No medication use | 0.96 (0.60-1.56) | 0.883 | 1.04 (0.63-1.73) | 0.872 |

^a)^**Model M3:** Adjusting for sex, age, health insurance scheme, geographic region, hospital level, T2D comorbidity, BMI, HTN duration, smoking status, control BP, and number of anti-hypertensive medication use.

^b)^**Model M4:** Using marginal structural models, and adjusting for sex, age, health insurance scheme, geographic region, hospital level, T2D comorbidity, BMI, HTN duration, smoking status control BP, and number of anti-hypertensive medication use.

ECG-LVH: Electrocardiographic left ventricular hypertrophy, AOR: adjusted odds ratio, CI: confidence interval

The variables of antihypertensive medication were available on the dataset in 2014, 2015, and 2018; thus, the total N would be reduced from 38,807 (primary analysis) to 17,963.

**Table** **S6.** Associated between age and ECG-LVH in men and women (Model M5 and Model M6); added number of antihypertensive medications use

| **Factors** | **Men** | | **Women** | |
| --- | --- | --- | --- | --- |
|  | **AOR (95% CI)** | ***p* - value** | **AOR (95% CI)** | ***p* - value** |
| **Model M5** ^a)^ |  |  |  |  |
| **Age, years** |  |  |  |  |
| 20-49 | Ref. |  | Ref. |  |
| 50-59 | 0.83 (0.54-1.27) | 0.387 | 1.05 (0.60-1.81) | 0.873 |
| 60-69^†^ | 0.85 (0.56-1.29) | 0.455 | 1.90 (1.14-3.18) | 0.014 |
| 70-79^†^ | 0.99 (0.65-1.52) | 0.963 | 2.14 (1.27-3.61) | 0.004 |
| ≥80^†^ | 1.00 (0.61-1.64) | 0.995 | 3.60 (2.10-6.15) | <0.001 |
| **Model M6** ^b)^ |  |  |  |  |
| **Age, years** |  |  |  |  |
| 20-49 | Ref. |  | Ref. |  |
| 50-59 | 0.80 (0.52-1.23) | 0.312 | 1.03 (0.57-1.84) | 0.931 |
| 60-69^†^ | 0.83 (0.55-1.26) | 0.387 | 1.98 (1.13-3.46) | 0.017 |
| 70-79^†^ | 0.97 (0.64-1.48) | 0.905 | 2.12 (1.21-3.74) | 0.009 |
| ≥80^†^ | 0.98 (0.60-1.59) | 0.933 | 3.76 (2.11-6.70) | <0.001 |

^a)^**Model M5:** Adjusting for age, health insurance scheme, geographic region, hospital level, T2D comorbidity, BMI, HTN duration, smoking status, control BP, and number of anti-hypertensive medication use.

^b)^**Model M6:** Using marginal structural models, and adjusting for age, health insurance scheme, geographic region, hospital level, T2D comorbidity, BMI, HTN duration, smoking status control BP, and number of anti-hypertensive medication use.

ECG-LVH: Electrocardiographic left ventricular hypertrophy, AOR: adjusted odds ratio, CI: confidence interval

^†^*P* for interaction <0.05

The variables of antihypertensive medication were available on the dataset in 2014, 2015, and 2018; thus, the total N would be reduced from 38,807 (primary analysis) to 17,963.

**Table** **S7.** Multivariable analysis for factors associated with ECG-LVH (Model M7 and Model M8); added antihypertensive medications use categories

| **Factors** | **Model S7** ^a)^ | | **Model S8** ^b)^ | |
| --- | --- | --- | --- | --- |
|  | **AOR (95% CI)** | ***p*- value** | **AOR (95% CI)** | ***p*- value** |
| **Sex** |  |  |  |  |
| Women | Ref. |  | Ref. |  |
| Men | 1.34 (1.13-1.59) | 0.001 | 1.38 (1.16-1.65) | <0.001 |
| **Age, years** |  |  |  |  |
| 20-49 | Ref. |  | Ref. |  |
| 50-59 | 0.92 (0.66-1.28) | 0.623 | 0.90 (0.64-1.26) | 0.535 |
| 60-69 | 1.22 (0.89-1.68) | 0.219 | 1.25 (0.90-1.72) | 0.183 |
| 70-79 | 1.39 (1.01-1.93) | 0.045 | 1.38 (0.99-1.92) | 0.054 |
| ≥80 | 1.90 (1.35-2.70) | <0.001 | 1.97 (1.38-2.80) | <0.001 |
| **Health insurance scheme** |  |  |  |  |
| Universal health coverage | Ref. |  | Ref. |  |
| Civil servant medical benefit | 0.74 (0.61-0.89) | 0.002 | 0.72 (0.59-0.88) | 0.001 |
| Social security | 1.29 (0.88-1.89) | 0.202 | 1.30 (0.89-1.90) | 0.165 |
| Others | 0.86 (0.35-2.13) | 0.740 | 0.71 (0.27-1.89) | 0.494 |
| **Regions** |  |  |  |  |
| Northeast | Ref. |  | Ref. |  |
| North | 1.62 (1.26-2.08) | <0.001 | 1.58 (1.22-2.04) | <0.001 |
| Central | 1.44 (1.13-1.84) | 0.003 | 1.44 (1.12-1.86) | 0.004 |
| South | 1.47 (1.12-1.94) | 0.007 | 1.46 (1.10-1.93) | 0.009 |
| **Hospital levels** |  |  |  |  |
| Community hospital | Ref. |  | Ref. |  |
| Provincial hospotal | 1.54 (1.29-1.82) | <0.001 | 1.54 (1.29-1.85) | <0.001 |
| Regional hospital | 2.48 (1.94-3.18) | <0.001 | 2.81 (2.17-3.63) | <0.001 |
| **Type 2 diabetes comorbidity** |  |  |  |  |
| No | Ref. |  | Ref. |  |
| Yes | 1.05 (0.89-1.25) | 0.547 | 1.12 (0.94-1.34) | 0.198 |
| **Smoking status** |  |  |  |  |
| Never | Ref. |  | Ref. |  |
| Ex-smoker | 1.15 (0.92-1.43) | 0.231 | 1.16 (0.92-1.47) | 0.205 |
| Current smoker | 1.54 (1.10-2.15) | 0.011 | 1.53 (1.08-2.16) | 0.016 |
| **Hypertension duration, years** |  |  |  |  |
| 1-9 | Ref. |  | Ref. |  |
| 10-19 | 1.14 (0.95-1.36) | 0.178 | 1.13 (0.93-1.37) | 0.213 |
| ≥20 | 1.77 (1.01-3.12) | 0.047 | 1.55 (0.85-2.72) | 0.159 |
| **Body mass index, kg/m^2^** |  |  |  |  |
| <30 | Ref. |  | Ref. |  |
| ≥30 | 0.85 (0.66-1.09) | 0.181 | 0.86 (0.66-1.12) | 0.271 |
| **Blood pressure, mmHg** |  |  |  |  |
| SBP <140 and DBP < 90 | Ref. |  | Ref. |  |
| SBP <140 and DBP ≥ 90 | 1.02 (0.64-1.62) | 0.927 | 1.03 (0.63-1.68) | 0.898 |
| SBP ≥140 and DBP < 90 | 1.28 (1.08-1.53) | 0.005 | 1.28 (1.07-1.53) | 0.007 |
| SBP ≥140 and DBP ≥ 90 | 1.51 (1.18-1.93) | 0.001 | 1.54 (1.19-1.99) | 0.001 |
| **Category of anti-hypertensive medication use** | |  |  |  |
| ACEI/ARB only | Ref. |  | Ref. |  |
| CCB only | 0.82 (0.61-1.11) | 0.208 | 0.89 (0.65-1.23) | 0.487 |
| 𝛽-blockers only | 0.97 (0.62-1.52) | 0.896 | 1.02 (0.63-1.63) | 0.947 |
| Diuretics only | 1.45 (0.93-2.27) | 0.104 | 1.52 (0.96-2.40) | 0.074 |
| ACEI/ARB + CCB | 0.72 (0.53-0.98) | 0.034 | 0.76 (0.55-1.05) | 0.097 |
| ACEI/ARB + 𝛽-blockers | 1.59 (1.14-2.22) | 0.007 | 1.80 (1.26-2.56) | 0.001 |
| ACEI/ARB + Diuretics | 1.85 (1.31-2.61) | <0.001 | 1.96 (1.37-2.81) | <0.001 |
| CCB + 𝛽-blockers | 1.31 (0.91-1.88) | 0.142 | 1.34 (0.92-1.96) | 0.125 |
| CCB + Diuretics | 1.24 (0.80-1.92) | 0.340 | 1.24 (0.78-1.96) | 0.366 |
| 𝛽-blockers + Diuretics | 1.13 (0.62-2.05) | 0.688 | 1.13 (0.61-2.09) | 0.696 |
| ACEI/ARB + 𝛽-blockers + CCB | 1.57 (1.15-2.14) | 0.004 | 1.66 (1.19-2.30) | 0.003 |
| Others | 1.68 (1.29-2.18) | <0.001 | 1.78 (1.35-2.36) | <0.001 |

^a)^**Model M7:** Adjusting for sex, age, health insurance scheme, geographic region, hospital level, T2D comorbidity, BMI, HTN duration, smoking status, control BP, and category of anti-hypertensive medication use.

^b)^**Model M8:** Using marginal structural models, and adjusting for sex, age, health insurance scheme, geographic region, hospital level, T2D comorbidity, BMI, HTN duration, smoking status control BP, and category of anti-hypertensive medication use.

ECG-LVH: Electrocardiographic left ventricular hypertrophy, AOR: adjusted odds ratio, CI: confidence interval

The variables of antihypertensive medication were available on the dataset in 2014, 2015, and 2018; thus, the total N would be reduced from 38,807 (primary analysis) to 17,963.

Table **S8** Associated between age and ECG-LVH in men and women (Model M9 and Model M10); added antihypertensive medications use categories

| **Factors** | **Men** | | **Women** | |
| --- | --- | --- | --- | --- |
|  | **AOR (95% CI)** | ***p* - value** | **AOR (95% CI)** | ***p* - value** |
| **Model M9** ^a)^ |  |  |  |  |
| **Age, years** |  |  |  |  |
| 20-49 | Ref. |  | Ref. |  |
| 50-59 | 0.84 (0.55-1.29) | 0.423 | 1.03 (0.59-1.79) | 0.915 |
| 60-69^†^ | 0.84 (0.56-1.28) | 0.425 | 1.85 (1.11-3.1) | 0.019 |
| 70-79^†^ | 0.97 (0.64-1.5) | 0.907 | 2.09 (1.24-3.52) | 0.006 |
| ≥80^†^ | 0.96 (0.58-1.57) | 0.862 | 3.5 (2.05-5.99) | <0.001 |
| **Model M10** ^b)^ |  |  |  |  |
| **Age, years** |  |  |  |  |
| 20-49 | Ref. |  | Ref. |  |
| 50-59 | 0.82 (0.53-1.25) | 0.356 | 1.01 (0.56-1.81) | 0.975 |
| 60-69^†^ | 0.83 (0.55-1.25) | 0.366 | 1.93 (1.1-3.36) | 0.021 |
| 70-79^†^ | 0.96 (0.64-1.46) | 0.86 | 2.06 (1.17-3.62) | 0.012 |
| ≥80^†^ | 0.93 (0.57-1.52) | 0.784 | 3.66 (2.05-6.52) | <0.001 |

^a)^**Model M9:** Adjusting for age, health insurance scheme, geographic region, hospital level, T2D comorbidity, BMI, HTN duration, smoking status, control BP, and category of anti-hypertensive medication use.

^b)^**Model M10:** Using marginal structural models, and adjusting for age, health insurance scheme, geographic region, hospital level, T2D comorbidity, BMI, HTN duration, smoking status control BP, and category of anti-hypertensive medication use.

ECG-LVH: Electrocardiographic left ventricular hypertrophy, AOR: adjusted odds ratio, CI: confidence interval

^†^*P* for interaction <0.05

The variables of antihypertensive medication were available on the dataset in 2014, 2015, and 2018; thus, the total N would be reduced from 38,807 (primary analysis) to 17,963.

**Table S9.** Sensitivity analysis for unmeasured confounding using E-value for odds ratio.

| **Factors** | **Primary analysis** ^a)^ | **E-value for OR** | |
| --- | --- | --- | --- |
|  | **AOR (95% CI)** | **Point estimate** | **CI** |
| **Sex** |  |  |  |
| Women | Ref. |  |  |
| Men | 1.49 (1.31-1.69) | 2.34 | 1.95 |
| **Age, years** |  |  |  |
| 20-49 | Ref. |  |  |
| 50-59 | 0.95 (0.73-1.24) | 1.29 | 1.00 |
| 60-69 | 1.31 (1.02-1.68) | 1.95 | 1.16 |
| 70-79 | 1.56 (1.20-2.02) | 2.50 | 1.69 |
| ≥80 | 2.10 (1.58-2.78) | 3.62 | 2.54 |
| **Health insurance scheme** |  |  |  |
| Universal health coverage | Ref. |  |  |
| Civil servant medical benefit | 0.73 (0.63-0.84) | 2.08 | 1.67 |
| Social security | 1.07 (0.77-1.46) | 1.34 | 1.00 |
| Others | 1.02 (0.52-2.01) | 1.16 | 1.00 |
| **Regions** |  |  |  |
| Northeast | Ref. |  |  |
| North | 1.58 (1.30-1.91) | 2.54 | 1.92 |
| Central | 1.62 (1.34-1.95) | 2.62 | 2.03 |
| South | 1.39 (1.12-1.72) | 2.13 | 1.49 |
| **Hospital levels** |  |  |  |
| Community hospital | Ref. |  |  |
| Provincial hospotal | 1.46 (1.27-1.67) | 2.28 | 1.86 |
| Regional hospital | 2.41 (2.01-2.89) | 4.25 | 3.44 |
| **Type 2 diabetes comorbidity** |  |  |  |
| No | Ref. |  |  |
| Yes | 0.89 (0.79-1.02) | 1.50 | 1.00 |
| **Smoking status** |  |  |  |
| Never | Ref. |  |  |
| Ex-smoker | 1.18 (0.98-1.41) | 1.64 | 1.00 |
| Current smoker | 1.26 (1.09-1.46) | 1.83 | 1.40 |
| **Hypertension duration, years** |  |  |  |
| 1-9 | Ref. |  |  |
| 10-19 | 1.19 (1.03-1.39) | 1.67 | 1.21 |
| ≥20 | 1.88 (1.22-2.91) | 3.17 | 1.74 |
| **Body mass index, kg/m^2^** |  |  |  |
| <30 | Ref. |  |  |
| ≥30 | 0.88 (0.72-1.08) | 1.53 | 1.00 |
| **Blood pressure, mmHg** |  |  |  |
| SBP <140 and DBP < 90 | Ref. |  |  |
| SBP <140 and DBP ≥ 90 | 1.00 (0.69-1.44) | 1.00 | 1.00 |
| SBP ≥140 and DBP < 90 | 1.36 (1.18-1.56) | 2.06 | 1.64 |
| SBP ≥140 and DBP ≥ 90 | 1.58 (1.30-1.92) | 2.54 | 1.92 |

^a)^Adjusting for sex, age, health insurance scheme, geographic region, hospital level, T2D comorbidity, BMI, HTN duration, smoking status, and control BP.

ECG-LVH: Electrocardiographic left ventricular hypertrophy, OR: odds ratio, CI: confidence interval

**Example for E-value interpretation:**

Association between sex and ECG-LVH was observed, adjusted odds ratio 1.49 (95% CI: 1.31-1.69).

The E-value for the point estimate is 2.34.

This E-value can be interpreted as follows: “*The observed odds ratio of 1.49 could be explained away by an unmeasured confounder that was associated with both the sex and the ECG-LVH by an odds ratio of 2.34-fold each, above and beyond the measured confounders, but weaker confounding could not do so*” [1]

The E-value for the lower confidence limit is 1.95, which can be interpreted as “*unmeasured confounders associated with sex and ECG-LVH by a odds ratio of 1.95-fold each could explain away the lower confidence limit, but weaker confounding could not*”.[1]

**References**

1. Linden A, Mathur MB, VanderWeele TJ. Conducting sensitivity analysis for unmeasured confounding in observational studies using E-values: The evalue package. Stata Journal. 2020;20.
